# An imported enteric fever caused by a quinolone-resistant *Salmonella typhi*

**DOI:** 10.4103/0256-4947.65267

**Published:** 2010

**Authors:** Ali M. Somily

**Affiliations:** From the Department of Pathology-Microbiology, King Saud College of Medicine, Riyadh, Saudi Arabia

## Abstract

Recent reports indicate that nalidixic acid susceptibility correlates well with the clinical outcome of patients with *Salmonella Typhi* infection treated with quinolones. We report a case of enteric fever caused by *S Typhi* in which the isolate was resistant to nalidixic acid, but showed in vitro susceptibility to ciprofloxacin. Following treatment with ciprofloxacin, the clinical outcome was not satisfactory and the patient had a relapse. However, after using a higher dose of ciprofloxacin, the patient was cured. We recommend that all *Salmonella* systemic infections resistant to nalidixic acid with in vitro but decreased susceptibility to fluoroquinolones be treated with other antibiotics like third-generation cephalosporins or azithromycin. These patients should be closely followed up and observed for further relapse.

Typhoid fever is an acute, invasive, generalized infection of the reticuloendothelial system caused by *Salmonella enterica* subspecies *enterica* serotype typhi (formerly, *S typhi*), a similar but less severe disease can be caused by S Paratyphi A, B and C. It is a major cause of morbidity and mortality worldwide, estimated to cause more than 16 million illnesses and 600 000 deaths annually.^1^

Due to the increasing resistance to traditional antibacterial agents used for therapy (ampicillin, amoxicillin, co-trimoxazole and chloramphenicol), fluoroquinolones, such as ciprofloxacin and ofloxacin became the drugs of choice for the treatment of these infections.^2^ A number of resistance mechanisms to quinolones in certain parts of the world led to decreased susceptibility of *S Typhi* to these agents have been identified. Physicians treating patients infected with such organisms should use the previous travel destination to decide the most appropriate empiric antimicrobial therapy. In the present report we describe a case of quinolone-resistant typhoid fever diagnosed at King Khalid University Hospital, Riyadh, Saudi Arabia. The case was apparently imported from an area where such resistant *S Typhi* is present. We believe that this case should alert the microbiologist, medical practitioners and public health officials to the possibility of importation and spread of such infections.

## CASE

A 23-year-old Indian nurse presented in the emergency room at King Khalid University Hospital (KKUH) with high grade fever. The patient had arrived from India 3 months prior to her presentation with the high-grade fever and generalized colicky abdominal pain. She gave a history of sweating and rigors mostly at night. A diagnosis of typhoid fever was made in India for which she was treated with an oral cephalosporin (generation not specified) for 12 days. On examination the only positive signs were palor, dehydration, a temperature of 38.5°C, a pulse rate of 120 ppm and a blood pressure of 107/57 mm Hg. On admission, specimens for blood and stool cultures were taken. The patient was empirically started on ciprofloxacin. 500 mg orally twice daily.

*S Typhi* was isolated from the blood culture, but no pathogens were isolated from stool. The isolate was susceptible to ciprofloxacin with a mimium inhibitory concentration (MIC) of < 1μg/mL by the MicroScan automated system (MicroScan, Dade Behring, Siemens Deerfield, IL, USA, *www.medical.siemens.com*). After two days of treatment the patient showed some clinical improvement and was discharged home on ciprofloxacin 500 mg orally twice daily for seven days. Two months later the patient presented again with similar symptoms and a blood culture showed *Salmonella Typhi*. Susceptibility this time using E-test susceptibility testing (AB Biodisk Solna, Sweden, *www.abbiodisk.com*) showed an MIC of 0.125 mg/mL for ciprofloxacin and resistance to nalidixic acid (NA). According to microbiologist advice, the patient was started on ciprofloxacin 750 mg orally twice daily for 14 days. The patient was then discharged in good condition and no further relapses were encountered for the next 2 years.

## Discussion

This case presents several points of interest regarding the management of typhoid fever caused by *S Typhi*. Since it is non-invasive, blood culture is still the standard diagnostic method for invasive salmonellae enteric infection, but it is not as sensitive as bone marrow culture (60% to 80% versus more than 90%) since *S Typhi* is intracellular and there are few organisms in the blood.^2^–^4^ After the emergence of *S Typhi* strains resistant to the traditional first-line antityphoid drugs (ampicillin, chloramphenicol and trimethoprim-sulfamethoxazole [TMP-SMX]) in the last two decades, ciprofloxacin became and is still the most effective treatment for typhoid fever.^2^ However, the emergence of resistant strains, the potential toxic effects in children plus cost considerations limited the widespread use of ciprofloxacin as an alternative to the traditional first-line drugs.^5^–^9^ Antimicrobial resistance of *S Typhi* was rare prior to the 1980s. Chloramphenicol was the treatment of choice, but in the 1980s, resistance to chloramphenicol and alternative agents began to emerge in countries where *S Typhi* was endemic.^2^ Thus, ciprofloxacin became and is still the most effective treatment for typhoid despite limitations to widespread use. Strains of *S Typhi* resistant to chloramphenicol, ampicillin, and TMP-SMX were reported in South America, the Indian subcontinent, Africa and Saudi Arabia.^10^,^11^ At the same time, in southern Vietnam multidrug resistance became established by early 1990.^12^

Because of widespread dissemination of multi-drug resistant strains, ciprofloxacin became the drug of choice for typhoid fever. Shortly isolates of *S Typhi* with reduced susceptibility to quinolones began to appear from these same areas with resistance to first-line drugs, there by threatening the efficacy of these agents for treatment of typhoid fever.^10^

Strains of *S Typhi* with decreased susceptibility to ciprofloxacin (MIC ≥0.125 μg/mL) and resistant to nalidixic increased to 23% in the UK in 1999 mainly in patients returning from travel to Asia who did not respond well to treatment with fluoroquinolone antimicrobials.^11^ There has been also a recent emergence of drug resistant enterica serotype typhimurium (*S typhimurium*) phage type 104 (DT 104) which was resistant to at least five drugs, i.e., ampicillin, chloramphenicol, streptomycin, sulfonamide and tetracycline, and has become a predominant *salmonella* type in many countries, including the US, UK, Germany, and France.^14^–^17^

The current MIC break points for Enterobacteriaceae (including *S enterica*) for ciprofloxacin are ≥4 μg/mL for resistance strains and ≤1 mg/mL for susceptible strains. The break points for nalidixic acid are ≥32 μg/mL for resistant and ≤16 mg/mL susceptible strains.^12^,^18^

In Mumbai, India, nalidixic acid resistance in *S Typhi* increased to 83% in 2000.^19^ Nalidixic acid-resistant, ciprofloxacin-susceptible *S Typhi* led to an outbreak in Tajikistan involving more than 6000 patients in 1997.^20^ A similar increase of such resistant strains also appeared in the US, in which most of these strains were obtained by people travelling abroad, indicating the global spread of these strains.^15^ In the US, *S Typhi* resistance to nalidixic acid increased from 6.8% in 1996-1997 to 23.2% in 2000, indicating a global increase of resistance to nalidixic acid as (80%) of *S Typhi* in this country as acquired from abroad.^21^ The epidemic strain had a pulsed-field gel electrophoresis profile indistinguishable from that of isolates of multi-drug resistant Vi-phage type E1 from patients infected in India.^22^

The molecular basis of quinolone resistance in *S Typhi* is due to a non-transmissible spontaneously occurring point mutation in the chromosomal gene (gyr A, gyr B, par C, and par E). This point mutation altered enzymes (DNA gyrase and topoisomerase IV) that are targets for quinolone drugs. Other mechanisms of resistance like altered permeability of bacterial cell membranes and efflux pumps are not well understood yet.^23^,^24^ Recently a multi-drug resistant transferable plasmid-mediated resistance to quinolones via the qnr gene was discovered.^25^

Scattered plots of MIC of nalidixic acid compared with those of ciprofloxacin for *S Typhi* showed a bimodal peak distribution of nalidixic acid at ≤4 μg/mL and 256 mg/mL.^26^ Nalidixic acid-resistant salmonellae tend to have MICs for ciprofloxacin that cluster within the upper part of the current susceptibility range for ciprofloxacin (0.125-0.5 mg/mL) whereas nalidixic acid-susceptible salmonellae tend to have MICs for ciprofloxacin of ≤0.03 μg/mL. Nalidixic acid susceptibility of salmonellae has been regarded as a good screening test for reduced salmonellae susceptibility to fluroquinolones, which has high sensitivity and specifity for detection of salmonellae with reduced susceptibility to ciprofloxacin (MIC ≤ 0.125 mg/mL).^27^

The ratio of serum peak antimicrobial to MIC and ratios of 24-hour area under the serum concentration versus time curve (AUC) to MIC are major activity measres for fluoroquinolones.^28^,^29^ Twenty-four-hour AUC/MIC ratios of ≥100 are required to produce survival rates approaching 100% in experimental animal infection infected with salmonellae^30^ and AUC/MIC ratios of ≥125 have been associated with satisfactory outcome of fluoroquinolones among seriously ill patients with enteric fever.^31^ Peak/MIC ratios of 8-10 prevent the emergence of resistant mutants during fluoroquinolone therapy.^32^,^33^ Ciprofloxacin with MIC of 0.5 μg/mL using a standard adult dose of ciprofloxacin for adult 500 mg orally twice daily will result in peak/MIC ratio=5 and AUC/MIC=46, which is suboptimal for treatment.^34^

An outbreak of *S typhimurium* DT104 In Denmark causing enteric disease in 25 culture-confirmed cases, was reported in which 4/5 patients failed therapy and continued to have persistent diarrhea despite treatment with ciprofloxacin or ofloxacin; the strain was resistant to nalidixic acid and had reduced susceptibility to the fluoroquinolones.^35^ There have been at least two reports of development of resistance during treatment and illness recurred in both patients.^36^ *S typhimurium* and the original isolate of *S typhimurium* had a ciprofloxacin MIC of 0.03 μg/mL, and then after treatment with ciprofloxacin, the post-therapy ciprofloxacin MIC increased to 0.5 μg/mL at 10 days and and 2 μg/mL at 14 days, respectively, none and both patients failed initial treatment.^37^

Fever usually disappears faster in patients who are infected with a nalidixic acid-susceptible strain and about one-third of patients with nalidixic acid-resistant strains required re-treatment compared with < 1% infected with susceptible strains.^13^ In India, due to the increase in the number of patients failing ciprofloxacin monotherapy since 1997, the recommended therapy for enteric fever is ciprofloxacin 750 mg twice daily for 7 days in this situation.^22^ In Saudi Arabia there were handful of reports indicating the emergence of nalidixic acid resistance in Salmonella spp.^39^–^42^ The prevalence of nalidixic acid resistant Salmonellae in this country may be underestimated due to underreporting and non-routine testing of nalidixic acid testing in most of clinical laboratories in Saudi Arabia. In 1996, in a study from Riyadh, nalidixic acid resistance was 13% with a higher MIC to ciprofloxacin.^40^ In a 2004 study from the eastern part of Saudi Arabia, nalidixic acid resistance was 9.9% while ciprofloxacin resistance was 2.3% in non-typhoidal salmonellae.^40^ We recently reviewed all isolates of *Salmonella* spp (2007-2008) and found nalidixic acid resistance to be around 40% (unpublished data) with almost all of these resistant isolates expressing a higher MIC for ciprofloxacin when compared with sensitive strains. Recently a case of *S Typhi* infection complicated by intestinal obstruction and mesenteric lymphadenitis in a Bangladeshi housewife was reported. These complications were thought to be due to a delay in reporting the isolate sensitivity to nalidixic acid, which masked the relative resistance to ciprofloxacin and consequently there was low dosing of this agent. However, the patient responded well to one week of therapy with azithromycin and a higher dose of ciprofloxacin.^41^

In severe cases of quinolone-resistant typhoid fever, patients should be treated with a third-generation cephalosporin parenterally for at least 10 days, or at least for 5 days after defervescence.^42^ Although fluoroquinolones at a higher dose of 20mg/kg/day for 14 days can be used in areas with low prevalence of quinolones resistant *S Typhi*, there are no randomized antibiotics trials reported in patients with severe typhoid fever.^43^

In summary, this case illustrates the clinical importance of the reduced fluoroquinolone susceptibility in *Salmonella* spp. We encourage all laboratories in Saudi Arabia to review their policy on detection of resistance and reporting of quinolone resistance on these isolates, according to standards of the Clinical and Laboratory Standards Institute. Repeated testing of antibiotic susceptibility is indicated in patients who fail to respond clinically to quinolones as this might indicate development of resistance due to single point mutation during treatment. In addition, there is an increasing possibility of fluoroquinolone-resistant strains being present in travelers returning from developing countries (e.g India subcontinent) where enteric fever is still endemic. Nalidixic acid testing is critical to detect the decrease in the susceptibility of these organisms to quinolones and should be one of the routine tested agents on these organisms in the clinical laboratory according to international standards. Finally, despite the response of our patient to high-dose quinolones, we do not recommend the generalization of this approach for all patients with nalidixic acid resistant *S Typhi* until there is a larger clinical trial to prove the clinical efficacy of high doses of these drugs in invasive infections.
