# errata

**Published:** 2010

Somily AM. An imported enteric fever caused by a quinolone-resistant *Salmonella typhi*. Ann Saudi Med [serial online] 2010 [cited 2010 Sep 5];30:313-6. Available from:http://www.saudiannals.net/text.asp?2010/30/4/313/65267

Incorrect *Salmonella* terminology was applied. The term *Salmonella* Typhi (and abbreviated form *S* Typhi) should have been used in the title and elsewhere, according to current nomenclature (http://www.bacterio.cict.fr/salmonellanom.html;AMA Manual of Style, 10th edition, Section 15.4.2).

